# Comprehensive intra/extracellular myocardial structural and functional characterization of hypertensive heart disease phenotypes

**DOI:** 10.1186/1532-429X-18-S1-P228

**Published:** 2016-01-27

**Authors:** Jonathan C Rodrigues, Antonio Matteo Amadu, Amardeep Ghosh Dastidar, Gergely V Szantho, Cattleya Godsave, Laura E Ratcliffe, Amy E Burchell, Emma C Hart, Mark Hamilton, Angus K Nightingale, Julian F Paton, Nathan E Manghat, Chiara Bucciarelli-Ducci

**Affiliations:** 1 grid.410421.20000000403807336CMR Unit, NIHR Cardiovascular Biomedical Research Unit, Bristol Heart Institute, Bristol, United Kingdom; 2 grid.11450.310000000120979138Department of Radiology, University of Sassari, Sassari, Italy; 3 grid.410421.20000000403807336Cardionomics Research Group, Bristol Heart Institute, Bristol, United Kingdom

## Background

The European Society of Cardiology recognizes different hypertensive heart disease (HHD) phenotypes. HHD can be classified into 4 left ventricular (LV) phenotypes by indexed LV mass, mass to volume ratio (M:V) and indexed end diastolic volume (EDV) (Table 1). All remodeling/hypertrophy phenotypes carry adverse cardiovascular prognosis, but the underlying mechanisms are incompletely understood. We investigated differences in intra/extracellular myocardial structure and function between phenotypes with T1 mapping and myocardial strain analysis.

**Table 1:** CMR definitions of patterns of hypertensive heart disease

	Indexed LV mass (g/m2)	Indexed EDV (ml/m2)	M/V (g/ml)
Normal LV	↔	↔	↔
LV remodeling	↔	↓	↑
Concentric LVH	↑	↔	↑
Eccentric LVH	↑	↑	↔

LV mass = left ventricular mass, EDV = end-diastolic volume, M/V = mass : volume ratio, LVH = left ventricular hypertrophy. LVM and EDV are indexed to body-surface area

## Methods

88 treated hypertensive patients (49 ± 14years, 57% male, SBP: 167 ± 30 mmHg, DBP: 96 ± 14 mmHg) underwent 1.5T CMR and were compared with 29 age- and sex-matched normotensive controls (47 ± 14years, 59% male, SBP: 128 ± 12 mmHg, DBP: 79 ± 10 mmHg).

Native and post-contrast T1 myocardial values were measured with a modified look-locker inversion-recovery sequence. Extracellular volume (ECV) and myocardial cell volume fractions were calculated. Circumferential strain values were estimated by voxel-tracking.

## Results

There was a gradient of increasing LV mass from normal, to remodeling, concentric LVH and eccentric LVH (70 ± 9 g/m^2^ vs 75 ± 10 g/m^2^ vs 108 ± 24 g/m^2^ vs 122 ± 30 g/m^2^, p < 0.05 respectively). This was due to step-wise increases in both: 1) indexed myocardial cell volume (eccentric LVH: 82 ± 20 ml/m^2^ vs concentric LVH: 77 ± 16 ml/m^2^ vs remodeling: 57 ± 9 ml/m^2^ vs normal: 51 ± 7 ml/m^2^, p < 0.05 respectively) and indexed interstitial volume (eccentric LVH: 33 ± 10 ml/m^2^ vs concentric LVH: 30 ± 10 ml/m^2^ vs remodeling: 19 ± 2 ml/m^2^ vs normal: 18 ± 3 ml/m^2^, p < 0.05 respectively).

Eccentric LVH had significantly impaired peak circumferential strain (-13 ± 5% vs concentric LVH: -16 ± 3% vs remodeling: -17 ± 3% vs normal: -18 ± 3% vs controls: -17 ± 3%, p < 0.05 respectively), with evidence of both peak systolic (-70 ± 20%/sec vs concentric LVH: -98 ± 20%/sec vs remodeling: -115 ± 38%/sec vs normal: -107 ± 28%/sec vs controls: -101 ± 13%/sec, p < 0.05 respectively) and peak diastolic strain rate impairment (65 ± 21%/sec vs concentric LVH: 82 ± 23%/sec vs remodeling: 90 ± 24%/sec vs normal: 102 ± 26%/sec vs controls: 101 ± 26%/sec, p < 0.05 respectively).

Despite similar BP severity as LVH, LV remodeling was associated with neither significant intracellular/interstitial expansion (native T1 1029 ± 45 ms vs 1024 ± 41 ms, P=0.67), nor myocardial dysfunction compared to normotensive controls.

## Conclusions

We comprehensively characterize, at the intra/extracellular myocardial level, structural differences between hypertensive phenotypes that are associated with functional consequences. We show that:

1) LVH, in particular eccentric LVH, is associated with significantly elevated myocardial cell volume, interstitial volume as well as myocardial systolic and diastolic strain impairment.

2) LV remodeling is associated with both normal myocardial structure and function.

Our results may help explain why eccentric LVH has poor prognosis. Our findings may have implications for future anti-hypertensive treatments.

**Figure 1:**
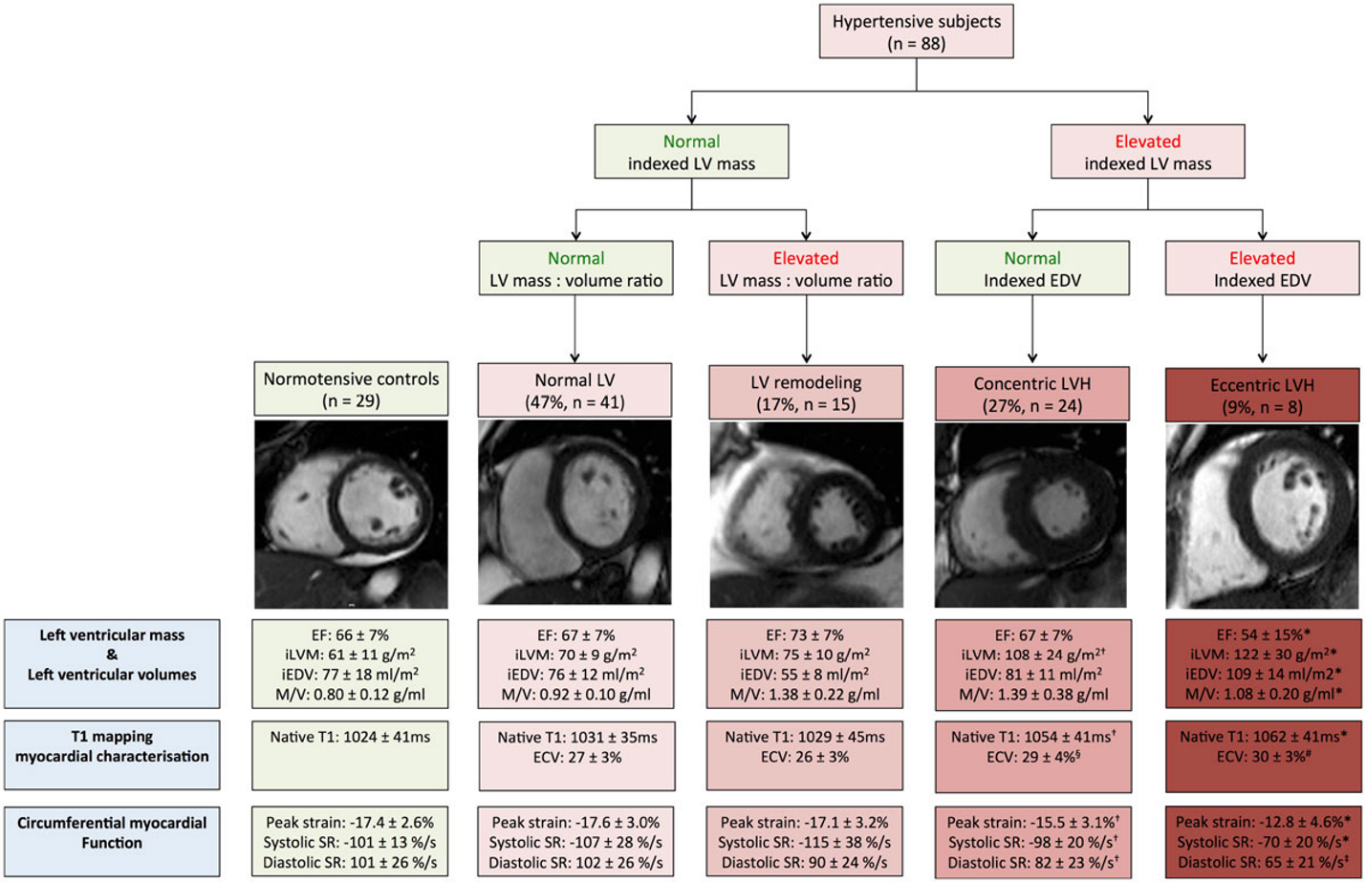
* P < 0.05, Eccentric LVH vs all subgroups. † P < 0.05, Concentric LVH vs LV remodeling vs normal LV vs controls. § P < 0.05, Concentric LVH vs LV remodeling. # P < 0.05, Eccentric LVH vs LV remodeling. ‡ P < 0.05, Eccentric LVH vs LV remodeling vs normal LV vs controls

